# Understanding variability in crop response to fertilizer and amendments in sub-Saharan Africa

**DOI:** 10.1016/j.agee.2016.05.012

**Published:** 2016-08-01

**Authors:** Job Kihara, Generose Nziguheba, Shamie Zingore, Adama Coulibaly, Anthony Esilaba, Vernon Kabambe, Samuel Njoroge, Cheryl Palm, Jeroen Huising

**Affiliations:** a International Center for Tropical Agriculture (CIAT), c/o ICIPE Duduville Complex, Off Kasarani Road, PO Box 823-00621, Nairobi, Kenya; b International Institute of Tropical Agriculture (IITA), c/o ICIPE Duduville Complex, Off Kasarani Road, PO Box 823-00621, Nairobi, Kenya; c International Plant Nutrition Institute, Sub-Saharan Africa Program, IFDC—East & Southern Africa Division, ICIPE Complex, Duduville-Kasarani, Thika Road, P.O. Box 30772-00100, Nairobi, Kenya; d Laboratoire Sol Eau Plante, Sotuba, Institut d’Economie Rurale, Rue Mohamed V, BP 262, Bamako, Mali; e Kenya Agricultural and Livestock Research Organization (KALRO), Kaptagat Road, Loresho Estate, P.O. Box 57811-00200, Nairobi, Kenya; f Lilongwe University of Agriculture and Natural Resources, Bunda College, PO Box 219, Lilongwe, Malawi; g Agriculture and Food Security Center, The Earth Institute, Columbia University, P. O. Box 1000, Palisades, NY, 10964, USA; h International Institute of Tropical Agriculture (IITA) PMB 5320, Oyo Road, Ibadan 200001, Oyo State, Nigeria

**Keywords:** Clustering, Nutrient omission trial, Crop responsiveness, Soil constraints, Variability

## Abstract

Improved understanding of soil fertility factors limiting crop productivity is important to develop appropriate soil and nutrient management recommendations in sub-Saharan Africa. Diagnostic trials were implemented in Kenya, Malawi, Mali, Nigeria and Tanzania, as part of the African Soils Information Service (AfSIS) project, to identify soil fertility constraints to crop production across various cropping systems and soil fertility conditions. In each country, one to three sites of 10 km × 10 km were included with each site having 12–31 field trials. The treatments tested included a control, an NPK treatment, three treatments in which the N, P and K nutrients were omitted one at a time from the NPK treatment, and three treatments in which secondary and micronutrients (Ca, Mg, S, Zn and B) simply referred here as multi-nutrients, manure and lime were added to the NPK. The field trials were conducted for 1–2 seasons; the test crop was maize except in Mali where sorghum was used. Nitrogen was limiting in all sites and generally the most limiting nutrient except in Sidindi (Kenya) and Kontela (Mali) where P was the most limiting. The general pattern in Kiberashi (Tanzania) shows none of the nutrients were limiting. K is mainly limiting in only one site (Mbinga) although incidences of K limitation were seen in almost all sites. Addition of multi-nutrients and manure further improved the yields of NPK in most sites. Cluster analyses revealed that maize crop in 11% of fields were highly responsive to nitrogen application, 25% (i.e., 21% poor and 4% fertile) ‘non-responsive’ to any nutrient or soil amendment, 28% being ‘low responsive’ and 36% of ‘intermediate response’. This study indicates that constraints to crop production vary considerably even within a site, and that addressing limitations in secondary and micronutrients, and increasing soil carbon can improve response to fertilizers. For sustainable crop production intensification in smallholder farming systems in SSA, there is need to develop management strategies to improve efficiency of fertilizer use and of other inputs, recognizing the site-specific nutrient response patterns at various spatial scales.

## Highlights

• In upto 25% of fields, maize is non-responsive to fertilizer and amendments.
• Multiple factors that vary by site explain poor crop response to fertilizers.
• Low Mn, Cu and B contents are the most striking differences between the poor non-responsive cluster and others.
• Site specific management recommendations are needed to improve the efficiency of fertilizer application.

## 1 Introduction

Achieving food security is a key agenda that is eluding governments in sub-Saharan Africa (SSA) (Shapouri et al., 2010). Low productivity of food crops due to low nutrient application, in a region that has faced land degradation for several decades, is one of the major contributors to food insecurity in SSA (IFDC, 2006, Shapouri et al., 2010, Muller et al., 2012), besides post-harvest losses and inequitable food distribution. The use of fertilizers remains very low in SSA (IFDC, 2006, Liu et al., 2010) despite the resolution to increase fertilizer use to 50 kg ha^−1^ by 2016 by the Africa Fertilizer Summit in 2006. Limited access and high costs of fertilizers are among the major causes of the limited use of fertilizers by smallholder farmers (Bumb et al., 2011). Nevertheless, there is an increase in fertilizer use in countries that are providing input subsidies such as Malawi, Mali, Nigeria and Tanzania (Sanchez et al., 2009, Druilhe and Barreiro-Hurlé, 2012), which is likely to increase further in the coming years. However, the fertilizer is often not targeted to specific crop, soil and agro-ecological conditions and application rates have for many years been based on blanket recommendations (Giller et al., 2011). The possible low response to fertilizer application as a result of this will likely frustrate efforts to increase fertilizer consumption. Information that can help to target the right fertilizer and application rates to the particular crop and location is crucial to improve the efficiency of the fertilizer use and for preventing negative environmental consequences.

Most research in SSA has focused on N and P as the key nutrients limiting crop production but there is growing evidence that other nutrients such as sulfur and some micronutrients constrain production (Weil and Mughogho, 2000, Nziguheba et al., 2009). Other factors that affect the efficiency of fertilizer use, such as soil acidity, also need to be taken into account. It has also become increasingly evident that, while crops respond favourably to N and P in some soils (so-called “responsive soils”), they do not respond to fertilizer application in any significant manner in other soils (the so-called non-responsive soils; Vanlauwe et al., 2011). In an analysis involving several agronomic trials for over 15 years, Kihara and Njoroge (2013) observed a large number of cases with low crop response to P. Two categories of soils where crops are non-responsive to fertilizers are defined: (i) soils in which low crop yields are observed and where crops respond poorly to fertilizers unless other amendments are applied (e.g. organic matter application, lime), and (ii) soils with high level of fertility and crops do not respond to nutrient application or soil amendments. Application of fertilizers to crops on both of these non-responsive soil categories result in very poor agronomic or economic efficiencies and in the former, also low water use efficiency (Kurwakumire et al., 2014). Although it is generally accepted that these crop fertilizer response classes exist, there is currently no information on their occurrence, extent, distribution or method of identifying them. The current study hypothesised that micronutrients are important in limiting crop productivity and are responsible for the non-responsiveness to macro-nutrients observed in SSA.

This study was conducted in a range of sites in SSA, in the context of the Africa Soil Information Service (AfSIS) project (www.africasoils.net), with three objectives: 1) to diagnose nutrients and other soil constraints that limit crop productivity in major cereal based cropping systems in SSA, 2) understand the prevalence and distribution of different classes of crop response to fertilizer, and 3) determine the soil nutrient related properties that characterize these classes of responses.

## 2 Materials and method

**Fig. 1:**
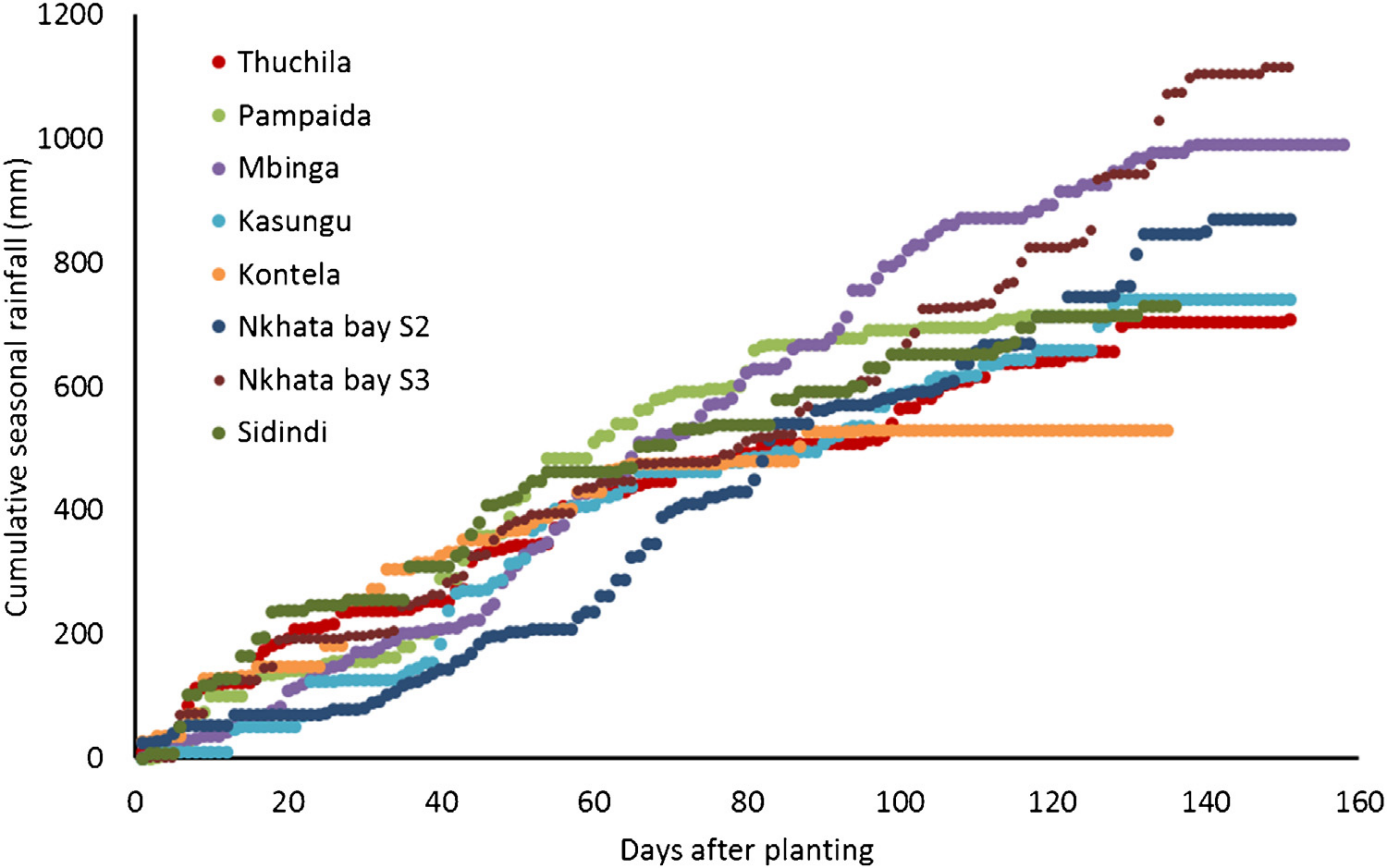
Cumulative rainfall as observed in the study sites.

Agronomic trials for identifying soil fertility constraints were implemented in Kenya (Sidindi in Western Kenya), Malawi (Thuchila in South, Kasungu in Central and Nkhata Bay in North), Mali (Kontela in Western and Koloko in Central), Nigeria (Pampaida in North) and Tanzania (Mbinga in South and Kiberashi in North). In each country, the 1–3 sites are predominantly agricultural, measuring an area of 10 km × 10 km and were chosen from the sentinel sites used in the AfSIS land degradation surveillance framework (LDSF), which had been identified using a random selection process. The sites were strategically selected to cover a wide range of biophysical conditions, ranging from semi-arid in northern Mali to more humid area in Tanzania, from fairly flat topographies of the Guinea Savanna in Nigeria to hilly sites in Malawi (Table 1 and Fig. 1).

Each 10 km × 10 km sentinel site had been divided into 16 equal blocks within which the field trials were conducted; ideally two fields in each of the 16 blocks were selected for the trials (a total of 32 fields per site) with each field representing a replicate of the trial. In some cases, however, less than 32 fields were used per sentinel site due to various limitations including blocks falling in non-agricultural lands, limited accessibility for crop monitoring, vulnerability to crop destruction from livestock and wild animals or non-suitability for the considered crop. Field trials were conducted between 2009 and 2012 and data used are for 1 season in all sites except in Nkhata Bay (Malawi) where these are for 2 seasons. The trials were conducted on a total of 310 individual fields among the countries.

### 2.1 Treatments and management

The test crop used was either maize or sorghum based on the major staple crop grown in the area; maize for sites in Kenya, Tanzania, Nigeria and Malawi; sorghum for sites in Mali. Improved varieties recommended for the area were planted and while the varieties vary between the countries and sites, the same variety was maintained within a site.

The field trials were implemented using a modified nutrient omission trial design. The treatments included a control (“Co”, no nutrient added), an NPK treatment (“NPK”), three treatments with omission of N (“−N”), P (“−P”) and K (“−K”), respectively, from the NPK treatment, three treatments with addition of secondary and micronutrients (referred to as multi-nutrients [“+MN”]), or Manure (“+MA”) or lime (“+L”) were added to NPK (Table 2). The +L treatment was not included in Mbinga because of logistic constraints in obtaining lime, and in Pampaida because pH was known to be generally above 5.5. In each field, each treatment appeared once, except for the Co and NPK treatments which had two replicates.

The macronutrients were applied at 100 kg N ha^−1^, 30 kg P ha^−1^, and 60 kg K ha^−1^ for maize and 60 kg N ha^−1^, 20 kg P ha^−1^, and 30 kg K ha^−1^ for sorghum. Secondary and micronutrients, in the +MN treatment, were applied at 10 kg Ca ha^−1^, 5 kg Mg ha^−1^, 5 kg S ha^−1^, 3 kg Zn ha^−1^ and trace amounts of B. Nutrient application rates were assumed to be non-limiting for all the sites and the selected crop. Manure was applied at 10 t ha^−1^ on dry matter basis and lime at 500 kg ha^−1^. Nitrogen was applied in 3 splits; a quarter at planting and the remainder in two equal splits at 3 and at 6 weeks after emergence. These field trials were designed and managed by researchers. The trials were implemented in collaboration with national partners in each of the countries following standard best agronomic management practices. The detailed description of the implementation methods for these trials is reported in Huising et al. (2013) and is also accessible online (http://afsis-dt.ciat.cgiar.org). For each of the field trials, an area of 50 m × 5 m or 25 m × 10 m was delimited to accommodate 10 plots of 5 m × 5 m corresponding to the eight treatments and two replications of the NPK and Co treatments. Plant spacing was 0.75 m by 0.25 m for maize and 0.80 m by 0.50 m for sorghum.

Soil sampling was done at trial establishment before application of fertilizers and amendments. Soil samples were obtained from 4 points of each 5 m by 5 m plot based on a Y-frame methodology, and a composite sample taken at field level. The composite samples were analysed for major soil characteristics by wet chemistry except for carbon and N that were predicted from Near- infrared spectroscopy (NIR), using the ICRAF spectra prediction models. Available soil P, exchangeable Al, S, B, Mn, Cu, Zn, K, Ca, Mg, Na and Fe were analysed by wet chemistry based on Mehlich 3 extraction procedure (Mehlich, 1984), pH was determined in water while phosphorus sorption index (PSI) was determined using potassium di-hydrogen phosphate (KH_2_PO_4_) extract at the Crop Nutrition Laboratories in Nairobi. Exchangeable sodium ratio (ESR) was calculated from available soil parameters as:

$$ESR=\frac{\text{Exchangeable Na}}{Sum\;of\;exchangeable\;\;bases-exchangeable\;Na\;}\;$$

Soil texture was determined as water dispersed particles after four minutes of ultrasonification at the ICRAF laboratory in Nairobi.

### 2.2 Measurements

Crops were harvested at maturity in a net plot of 6.75 m^2^ for maize and 7.2 m^2^ for sorghum, i.e., constituting the 3 middle rows in each plot, leaving 1 m on each side of the row. All plants in the net plots were harvested and the total fresh weights of cobs/heads and stover measured. Five cobs/heads (1 large, 3 medium and 1 small) were selected as subsamples from which all the grains were taken for drying. The sub-sample grain were dried to constant weight either in an oven at 60 °C or air-dried depending on availability of an oven at the collaborating research institutes in each country. Grain yield was expressed on dry weight (12.5% moisture content) basis and used to analyse responses to treatments.

### 2.3 Data analyses

Analysis of response to treatments was undertaken at the level of a sentinel site, comparing the treatment effects in the 12–31 fields, using R software (version 2.14.1, Foundation for statistical computing, 2011). The analysis excluded fields where responses to nutrients were deemed to have been affected by drought e.g. the complete first season in Thuchila, Malawi (29 fields) or where harvesting procedure was not correctly followed e.g., in first season in Sidindi Kenya (23 fields). Besides these two sites, individual fields within a site where harvesting was done only for 12 plants following extensive damage by animals were also omitted (a total of 18 fields). Thus, data from a total of 240 remaining fields were analysed. We used the model Grain yield ∼ treatment + (1|block) + (1|field within block) to study the effect of the treatments on crop grain yield. Two random terms were used in the model, one being the block within the sentinel site and the second the field within the block. Where data were available for multiple seasons, an additional random term, “(1|Season)” was included. The resulting coefficients were extracted and used to estimate the effects of nutrient application or omission on yield, and these are presented in comparison to the NPK treatment to provide insight in the yield gain/loss when a nutrient was omitted or amendments were applied. These effects were calculated as Yt-Ynpk where, Yt is yield of the treatment under consideration, and Ynpk is average yield of the NPK treatment. Also, the treatment effects were regressed against environmental mean calculated as the mean yield for all treatments in a given site, following the stability analysis approach presented by Raun et al. (1993).

Clustering analysis helps to identify meaningful groups with similar characteristics within a dataset (Kaufman and Rousseeuw, 2009). We applied this concept to identify various classes of nutrient response patterns in an attempt to characterize them and ultimately define and target management of soil fertility. Cluster analysis was conducted on the differences between the grain yield from a given treatment and that from the control treatment using K-Means clustering. The control treatment was used as the reference to ensure that all fertilizer treatments, even the NPK, were included in the clustering. Since there was no way of accounting for crop type in the clustering, only sites with maize (a total of 192 fields) were included in the cluster analysis.

In order to define the number of appropriate clusters for treatment responses based on explained variance, cluster analyses (K-means) were conducted with 2–15 clusters and the amount of variation explained by the successive clusters determined. An elbow plot of the explained variance of the clustering model as a function of the number of clusters k was made (see also Zhao and Sandelin, 2012) and four final clusters selected. The final cluster analysis was then conducted with the selected number of clusters, and biplots of grain yield data in these clusters plotted in R. Based on these analyses, clusters with various degree of responsiveness to fertilizers and amendments were identified. A non-responsive cluster where yield of the control treatment was similar to the fertilizer treatments was observed. Because this non-responsive cluster contains both poor and fertile non-responsive fields, we separated it into poor (<3 t ha^−1^) and fertile (>3 t ha^−1^) non-responsive clusters. This cut-off, assumed here to be the maximum yield for non-response, corresponds to the first-stage overall “green revolution” yield target of 3 t ha^−1^ in sub-Saharan Africa (Sanchez, 2010) and only slightly higher than maximum in the range of 2.1–2.8 t ha^−1^ maize grain yields in non-responsive fertile fields reported in Kenya, Uganda, Zimbabwe, Tanzania and Mozambique by Tittonell and Giller (2013) under smallholder farmer fields. The grain yield from these clusters (separating poor and fertile non-responsive cases) was further analyzed using the model Grain yield ∼ Treatment +(1|site) + (1|block) + (1|field with block) + (1|season) to obtain cluster-based mean treatment yields.

A multinomial logit regression model was developed to identify the possible soil factors influencing allocation of a field to a specific cluster. We used multinomial logit (mlogit) library in R to run the model: cluster ∼ 1|K + pH + total carbon+ Al+ Mn+ S + Ca:Mg + sum of exchangeable bases+ B+ Fe+ P+ Cu+ Zn+ Na, and used the poor non-responsive cluster and also the low responsive cluster as references (base categories). Due to soils data missing for some of the fields (in Pampaida), a total of 162 fields were included in the multinomial logistic regression. For each of the clusters, the median, minimum and maximum soils values are presented to provide further insight into their characteristics.

## 3 Results

### 3.1 Soil characterization

Soil data from the specific experimental fields within a site showed wide variability in major properties with median soil pH ranging from 5.2 to 6.4, and the available phosphorus from 3.6 to 52.8 mg kg^−1^ (Table 3). All sites, except the three in Malawi (Kasungu, Nkhata Bay and Thuchila), had median available P far below the critical value for maize of 15 mg kg^−1^ (Nandwa and Bekunda, 1998). Soils in Mbinga and Sidindi were clayey, with clay content above 80% in most fields, whereas Kasungu and Pampaida were the sandiest soils with median sand content of at least 50%. Soil organic carbon was >1% in Kiberashi, Koloko, Mbinga and Sidindi and <1% in the sandy sites such as Pampaida, Kontela, Thuchila and Kasungu.

### 3.2 Response to treatments

**Fig. 2:**
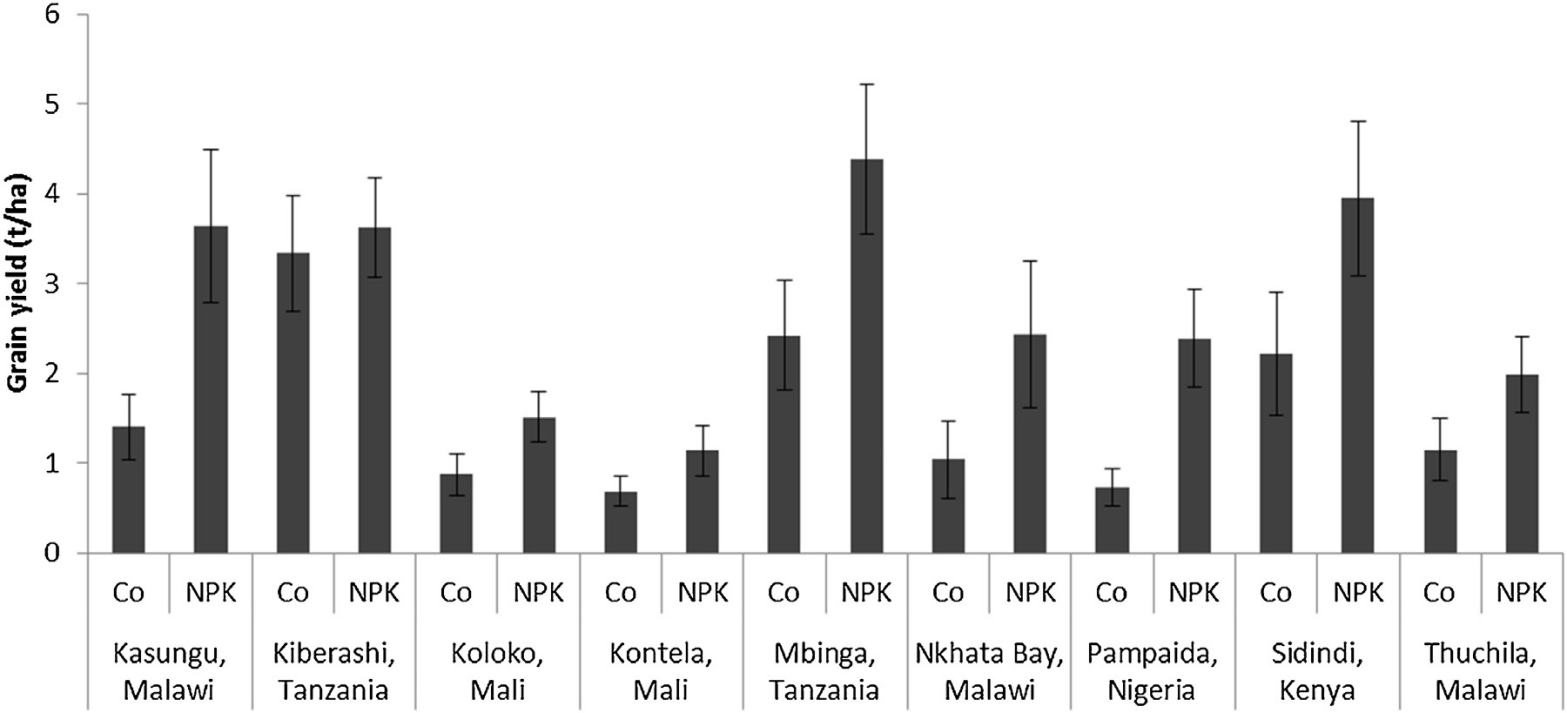
Observed grain yield in Control and NPK treatments in various sites. Sorghum was the crop in Koloko and Kontela while maize was in all the other sites. Error bars are standard deviations.

**Fig. 3:**
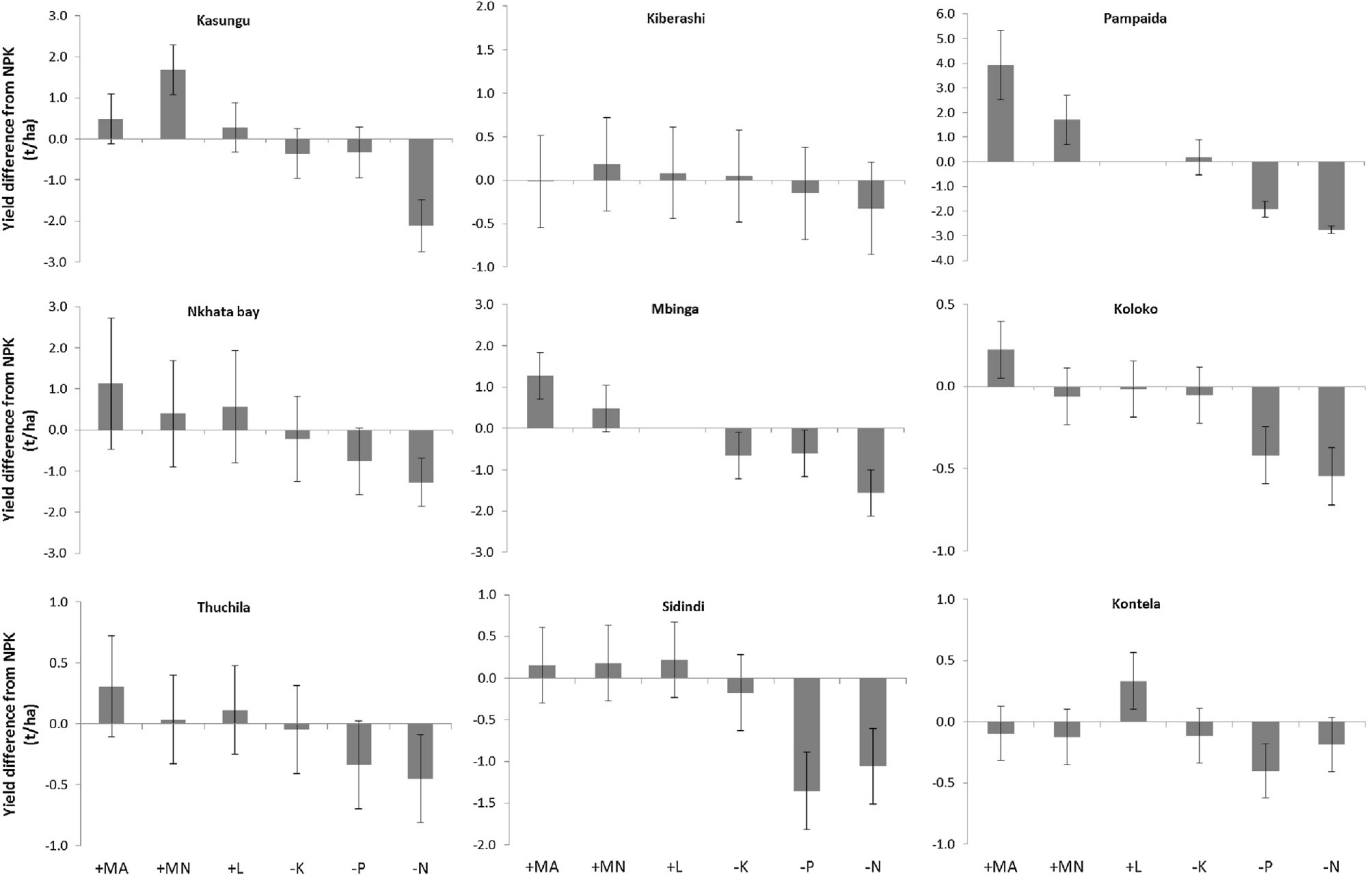
Effect of omission of macronutrients, lime and organic (manure) amendment and application of multi-nutrients on yield difference relative to NPK in selected AfSIS trial sites. Sorghum was the crop in Koloko and Kontela while maize was in all the other sites. Error bars are confidence intervals. Note: different y-axis scales used.

The overall effect of NPK application on crop yields varied among the various sites (Fig. 2). Average yields were significantly increased by the application of NPK in all sites except Kiberashi. A doubling of control yields was observed from the application of NPK in Kasungu, Nkhata Bay and Pampaida, whereas an increase of over 75% was obtained in Koloko, Mbinga and Sidindi, leaving only Thuchila and Kontela with an increase in NPK yield of less than 65%. In Kiberashi, none of the treatments, including manure and multi-nutrients increased the yields significantly above the control (Fig. 3); yields of the control plots in Kiberashi were about 3.5 t ha^−1^ on average. Highest NPK yields were obtained in Mbinga, where the average yield was 4.4 t ha^−1^.

There was a large difference between sites in the loss or gain in yield resulting from the omission of a macronutrient from the NPK treatment or addition of amendments to NPK (Fig. 3). Omission of N (the −N treatment) led to a reduction in yield compared to the NPK treatment in all sites, but this reduction was relatively low in Kiberashi, Kontela and Thuchila. All maize growing sites, except Kiberashi and Thuchila, encountered at least 1 t ha^−1^ reduction in yield following N omission, and the highest reduction of more than 2 t ha^−1^ on average was observed in Pampaida and Kasungu. An important observation was that, excluding the more recently reclaimed Kiberashi site, N is the dominant macronutrient limiting production in one site only (Kasungu), whereas in the other sites P is also limiting. Relatively large yield reductions, as a consequence of P omission (−P treatment) were observed in Pampaida, Sidindi, Koloko and Kontela. In Sidindi and Kontela, the yield reductions from −P were greater than those from the −N treatment.

Omission of Potassium (−K) resulted in significant and consistent yield reductions only in Mbinga. Addition of manure to NPK (+MA treatment) increased yields significantly in Pampaida, Mbinga, Nkhata Bay and Koloko, with an average gain of 4 t ha^−1^ in Pampaida. Significant yield gains from the addition of multi-nutrients (+MN treatment) were observed in Pampaida and Kasungu, with the gains from the latter site surpassing that from manure. In Mbinga and Nkhata Bay, especially, we find instances with significant gains in yield as a result of the application of multi-nutrients. Thuchila, Sidindi and Kiberashi also show incidental effect of the multi-nutrient application, though to lesser extent relative to Mbinga and Nkhata Bay. Addition of lime to NPK resulted in significant yield gains only in Kontela, with some fields in Nkhata Bay, Thuchila and Sidindi also showing response to lime. Within all the individual sites, there was considerable field to field variation in the response to the different nutrients and amendments, indicating pockets where specific factors are limiting.

**Fig. 4:**
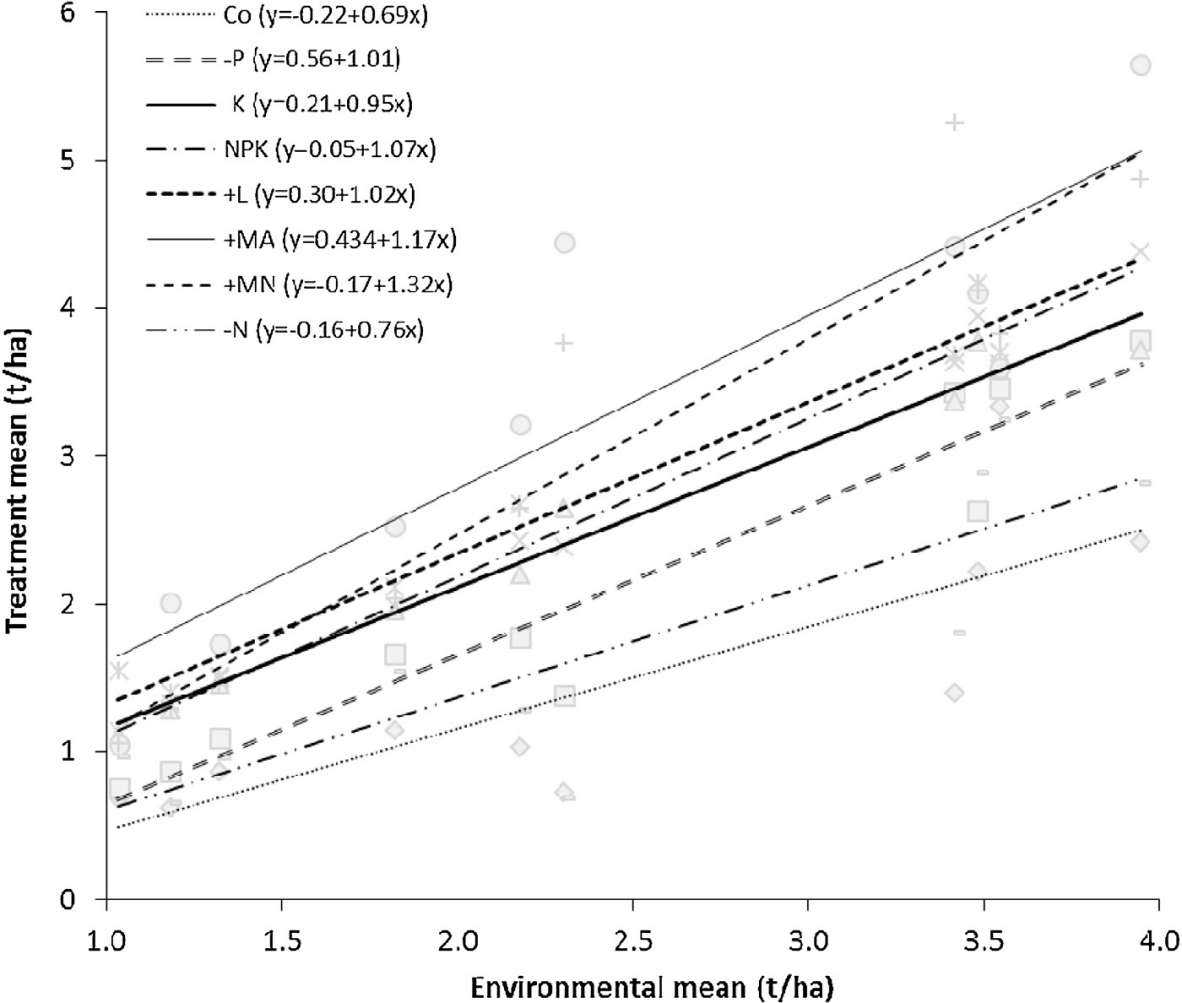
Regression of cereal grain yield in different treatments on the environmental mean for 9 sites used for field trials, 2009–2012.

From stability analysis (Fig. 4), three key observations can be made: 1) treatments without N (Co and −N) had the lowest intercept and smallest slope compared to the other treatments, 2) treatment where P is omitted (−P) gave a larger yield response in good environments compared to the treatment where N was omitted (−N); 3) the +MN treatment had superior response in high yielding environments when compared to all other treatments. Further, it is observed that treatment with manure (+MA) performed well in all environments.

### 3.3 Response clusters

**Fig. 5:**
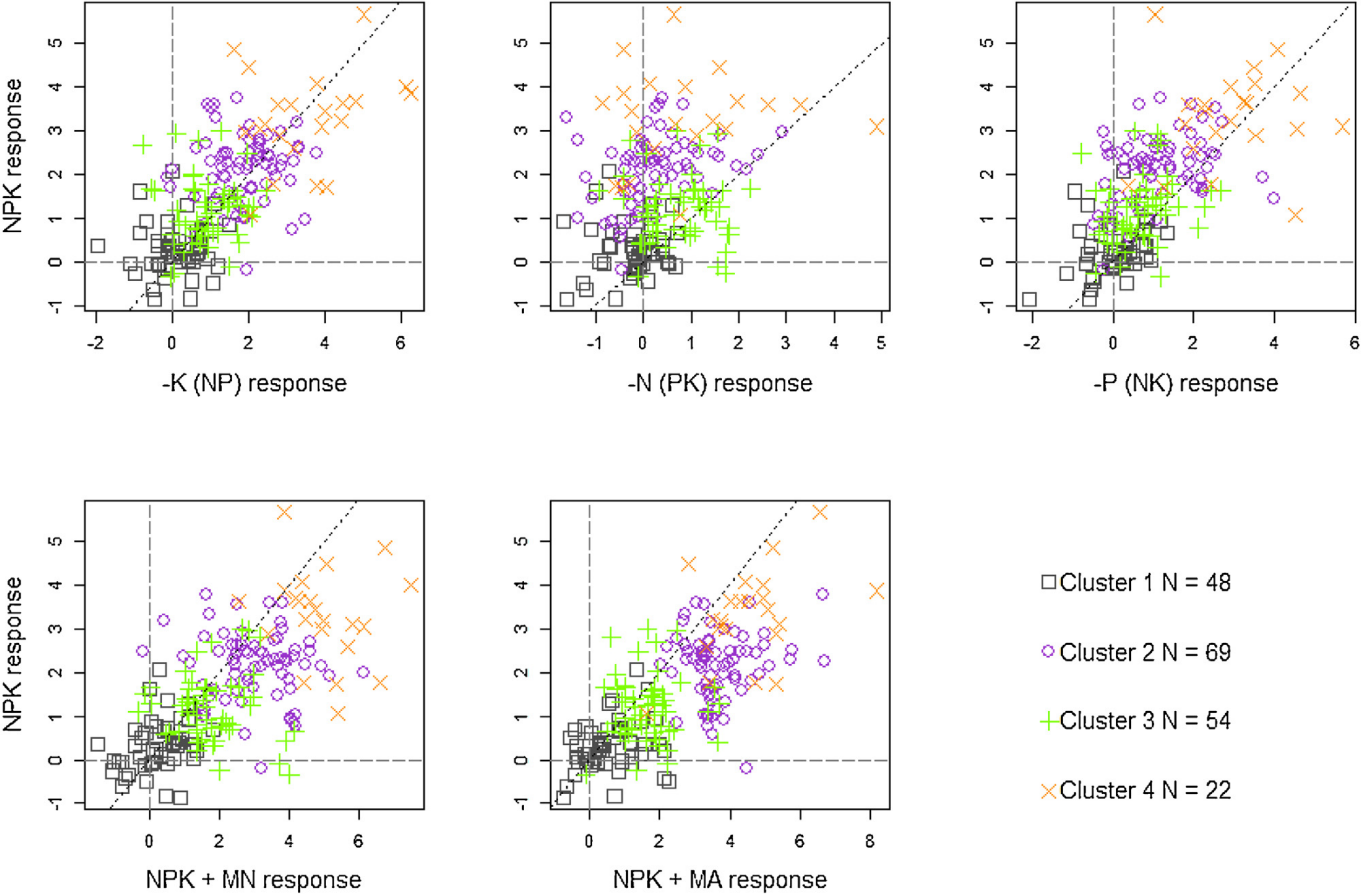
Plots of the resulting 4 clusters from the analysis of the diagnostic trial maize grain yield data. Treatment applied with lime is omitted since it was not applied in Mbinga and Pampaida sentinel sites. Longdash lines indicate where yield of control equals that of fertilizer treatment, dotted lines are 1:1 lines.

**Fig. 6:**
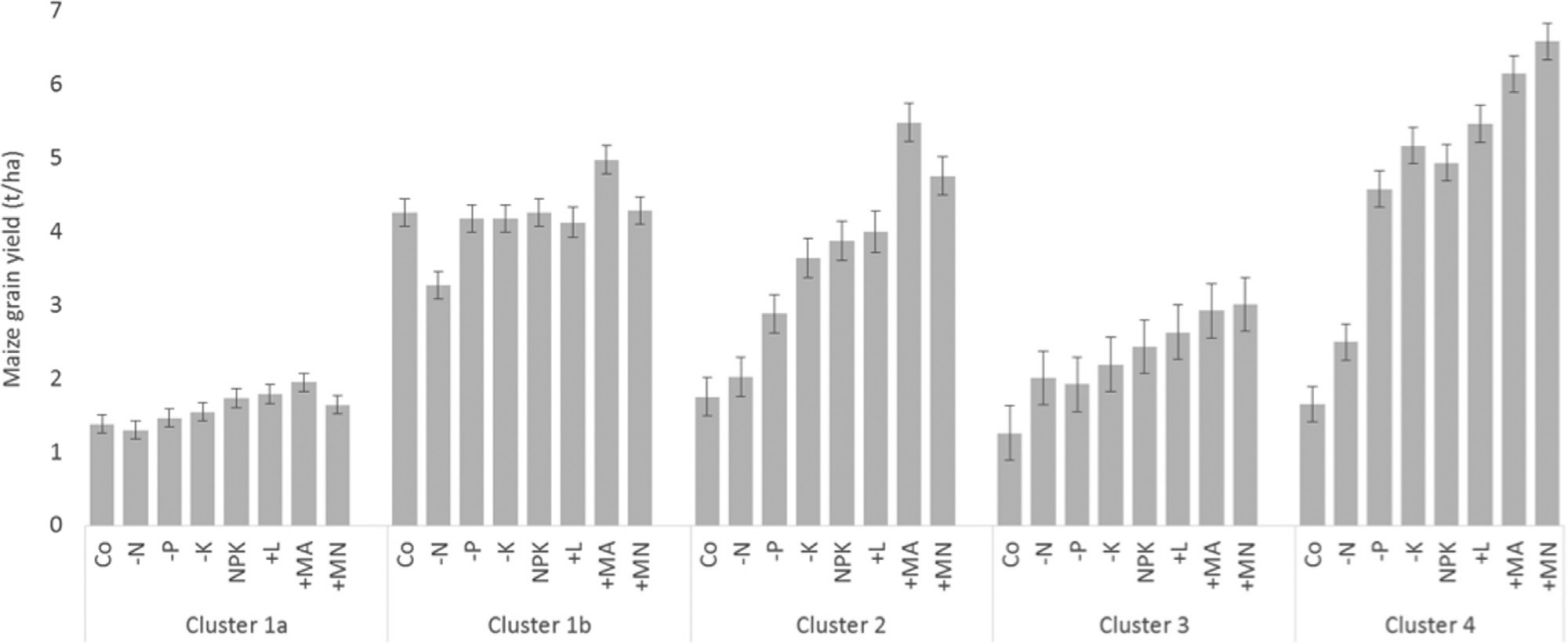
Maize grain yield observed from fields classified under different clusters following K-Means clustering. Error bars are standard errors of the estimates.

We selected 4 clusters as the appropriate number for our dataset because the extra amount of variation explained by an extra cluster was small (<5%). The four k-means clusters account for 60% of the variation in the yield data. By plotting the clusters of points in bi-plots of various combinations of response to the different treatments relative to the control treatments (Fig. 5) and the yields obtained from the various treatments (Fig. 6) the 4 clusters can be interpreted as follows:

• Cluster 1: Fields in which maize is not responsive to any nutrient application or soil amendments. Adding nutrients with or without amendments does not improve the yields. Some of these fields have fertile soils (cluster 1b in Fig. 6 referred to as fertile non-responsive fields) with high yields (attainable yield level between 4–5 t ha^−1^). Others are infertile with low yields (cluster 1a referred to as poor non-responsive fields, attainable yield level remains below 2 t ha^−1^) and have some limitations that need to be addressed before any nutrients or amendments can have an effect. About 25% of the fields considered in this study are in this non-responsive cluster (Table 4).
• Cluster 2: Fields with major N and P limitations and occasionally K limitations (combination of these nutrients is required to get at least 100% yield increase over the control). Addressing N, P and/or K limitations results in yields up to 4 t ha^−1^. Adding manure further improves the yield substantially (by 40% over NPK). Adding multi-nutrients to the NPK (i.e., the +MN treatment) also improves the yields significantly (by 23% over NPK). Attainable yield level with the proper inputs is around 5.5 t ha^−1^. In this cluster, it is clear that crop production is often constrained by a suite of factors including major nutrient limitations, requiring addition of organic matter. Thirty-five percent of the fields fell into this cluster. Fields in this cluster are referred to as highly responsive fields in the text.
• Cluster 3: Fields where maize has limited response to nutrient application and also limited response to further addition of amendments. While nutrients are required, these fields seem to have some other constraints that limit nutrient uptake and hence yield response. Attainable yield level is around 3 t ha^−1^. There are 28% of the fields that are in this poor response cluster, and are referred to as low responsive fields.
• Cluster 4: Fields with N as the major limiting factor and maize is highly responsive to N application but limited response to P and no clear further response to K. Addition of either lime, multi-nutrients or manure further improve the yield. Fields in this cluster constitute 11% of the cases and are referred to as highly N responsive fields. Attainable yield level with the appropriate macro-nutrient inputs is 5 t ha^−1^, but can be increased to 6.5 t ha^−1^ with the required soil amendments.

**Fig. 7:**
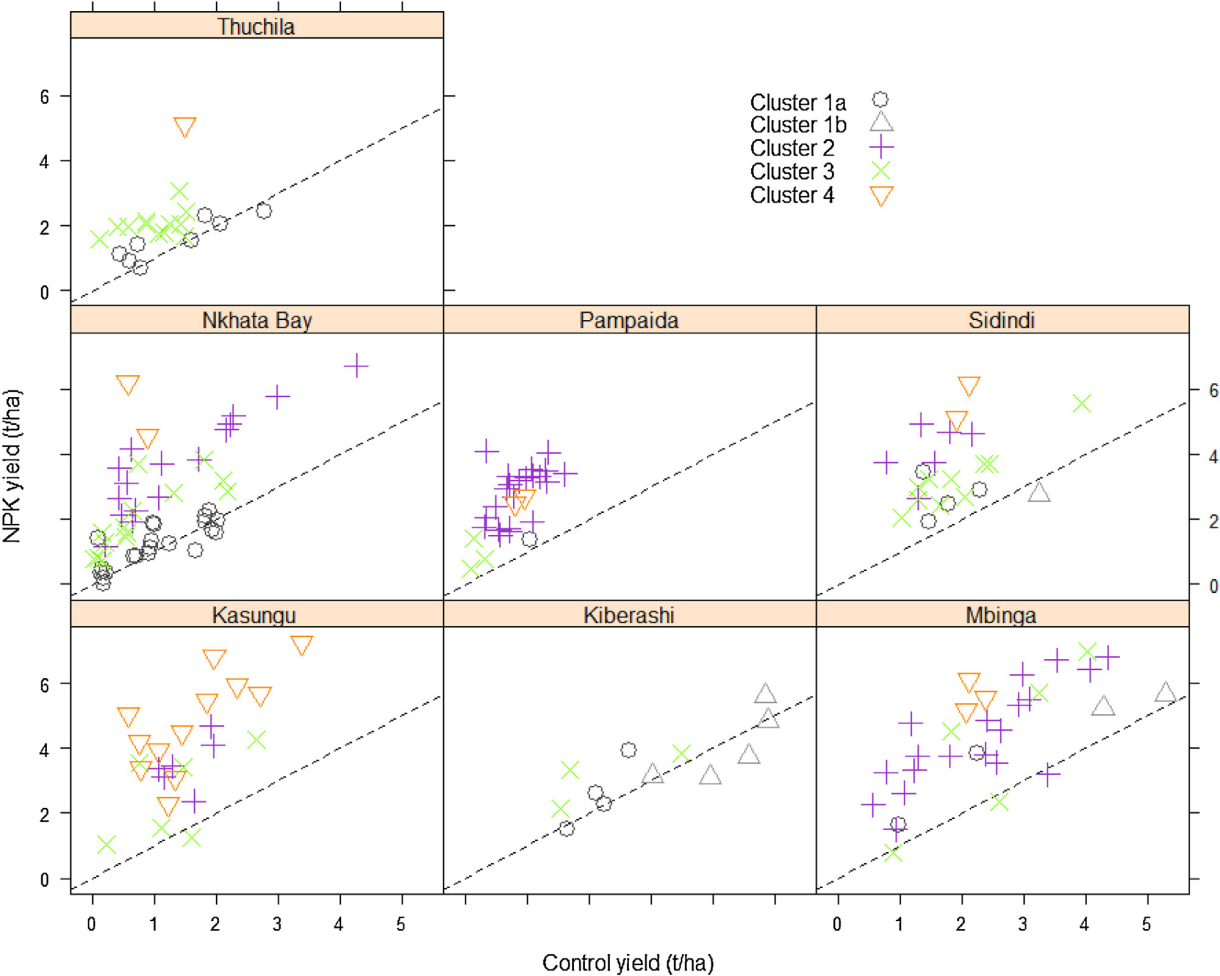
Maize grain yields of NPK in relation to those in the control treatments for the various clusters and sites. 1:1 lines are shown by the broken lines through each plot.

Sites vary in the response classes represented (Table 4, Fig. 7). Fifty percent of the fields in Kasungu belong to cluster 4, showing a very high maize response to N with yields in the NPK treatment reaching up to 7 t ha^−1^. The site does not have any field in the non-responsive category, but has some fields in the low and highly responsive categories. In contrast, Kiberashi, the site that was most recently cleared, has most of the fields in the fertile non-responsive category and some in the low response category. Control yields may reach up to 5 t ha^−1^, indicating high soil fertility. This type of non-responsiveness is a result of high fertility with little increase in yields from the application of nutrients or amendments.

Mbinga and Pampaida had most of the fields in the highly responsive category (cluster 2). The striking difference between the two sites is that Pampaida had relatively low control yields (less than 2 t ha^−1^ in all fields) and consequently relatively low NPK yields, whereas the control yields in Mbinga range from 0.6 to over 4 t ha^−1^, with corresponding higher NPK yields (Fig. 7). Similar to Pampaida, maize in Mbinga site responded to all nutrients including K. Most of the fields in Sidindi and Nkhata Bay are in the poor non-responsive and low response categories, but still with some fields in the highly responsive class. Thuchila has all but one field in the ‘non-responsive’ or ‘low response’ categories, and is characterized by very low yields among all maize sites with around 1 t ha^−1^ in the control treatment.

The fields in the poor non-responsive category (with yields <3 t ha^−1^) represent 21% of the fields – it is therefore important to identify the specific characteristics of such poor non-responsive fields to make targeted recommendations for restoring their productivity. Amongst the clusters, the non-responsive cluster had the lowest Zn, B, Cu, Mn and Na (Table 5). Using the poor non-responsive fields in cluster 1 as the base category in the multinomial logit shows that increasing soil Ca:Mg ratio is highly significant and increasing Zn, S, B and Na and simultaneously decreasing Al concentrations is significant (P < 0.05) in order to translate the poor non-responsive fields to the highly N responsive category of cluster 4. Increasing Zn, Mn and Al is significant (p < 0.01) to move the poor non-responsive fields to the highly responsive category of cluster 2 which is responsive to most of the nutrients and amendments. The poor non-responsive fields clearly had less carbon than the fertile non-responsive fields (1.4 vs 2.0%C, respectively) besides the limitations due to low B and exchangeable bases. Considering the low-responsive cluster 3 as the base category, increasing B and raising the Ca:Mg ratio would increase the probability of a field in this cluster belonging to the highly N responsive category (cluster 4).

## 4 Discussion

### 4.1 Diversity of crop responsiveness to nutrient and amendments

Wide variability in crop response to nutrients, manure and lime application was observed both within and between sites, reflecting a high degree of heterogeneity in soil characteristics and crop growing conditions at various spatial scales. This adds support to the need for tailoring soil fertility management practices to site-specific conditions to sustainably increase crop productivity in SSA (Giller et al., 2011, Vanlauwe et al., 2015). Three crop response categories that distinguish soils as responsive and non-responsive to fertilizer application (i.e. responsive, fertile non-responsive and degraded non-responsive) have often been used to simplify the complex yield response patterns that are characteristic of smallholder farms in SSA (Zingore et al., 2011, Tittonell et al., 2010). The analysis conducted in this study, based on a larger dataset, generated fertilizer response clusters that were consistent with the previous studies, but also show the need to disaggregate further the fields with low crop response and fields where crops are particularly responsive to application of N alone (Table 4; Fig. 6). Nitrogen deficiency is recognized as the most limiting nutrient in cereal crop production in large areas in SSA, and the identification of soils that predominantly respond to N application is relevant for developing management practices that optimize N use efficiencies (Vanlauwe et al., 2011). All the sites that were covered in this study have at least three of the five response classes represented indicating the large diversity in response classes within the sites (Table 5, Fig. 7).

Study sites in Kasungu, Mbinga and Pampaida stood out as having a high frequency of the fields where crops were more responsive to fertilizers, whereas other sites (Kiberashi, Nkhata Bay and Tuchila) were characterized by a majority of poor non-responsive and low response classes (Table 4; Fig. 7). The high prevalence of poor non-responsive and low responsive soils observed in this study indicate major challenges for increasing crop productivity, as attainable yields in more than 50% of these sites were less than the initial yield target of 3 t ha^−1^ towards achieving the African Green Revolution in SSA (Sanchez, 2010), even when nutrient and other agronomic inputs are applied in adequate quantities. Several studies have highlighted the challenge of the degraded soils where crops are non-responsive to fertilizer across different farming systems in SSA (Tittonell et al., 2007, Zingore et al., 2007, Vanlauwe et al., 2010, Kurwakumire et al., 2014), with estimates of degraded soils covering as high as 65% of the cropland (Vlek et al., 2008). Even though the results from this study are based on a relatively small number of sentinel sites, the overall high percentages of non and low responsive classes are consistent with other studies that show high prevalence of degraded and poor responsive classes in sub-Saharan Africa.

The non-responsiveness due to high fertility level observed in this study (cluster 1b; Fig. 6) requires fertilizer application only for maintenance purposes in the short-term. This type of non-responsiveness is mainly expected in areas newly converted to cultivation as in Kiberashi, or in fields close to homestead that receive large applications of fertilizer and manure (Giller et al., 2011, Zingore et al., 2008), and could represent a very small fraction of cases (4% in this study) of non-responsiveness to fertilizers in smallholder farming systems in SSA. One striking characteristic of this cluster is the high exchangeable bases compared to other clusters.

For the low-responsive class there is limited response to the treatments (NPK, and +MA and +MN especially), but the yield levels are far from the attainable yield levels observed within the respective sites (Fig. 6). This seems to suggest that the poor response is explained by soil conditions other than those that are remedied by the various treatments of the diagnostic trials. This is confirmed by the analyses of the soil characteristics in relation to the response classes.

### 4.2 Responsiveness and soil characteristics

Despite the importance of soil organic matter for soil fertility management in SSA, SOC was not a defining factor for the response classes, and there may be need to focus on C saturation deficit as an indicator of crop response (Six et al., 2002, Chivenge et al., 2007, Kimetu et al., 2009). For example, SOC contents were low for all the 5 response classes, with the median SOC values for the more responsive classes (clusters 2 and 4) even significantly lower than for the less responsive classes (Table 5).

The situation was different with respect to soil acidity. A relatively high pH and low Al concentration was associated with the highly responsive cluster (Table 5). These, together with Ca:Mg, are likely to be the distinguishing factors between the high and intermediate response categories. Nkhata Bay, and to a lesser extent Sindindi, show an overall strong, though highly varied, response to lime application. Both sites are characterized by low pH and high Al concentrations (though also very variable) and strong presence of non-, low and intermediate response classes (Table 3, Table 4).

There is growing evidence that micronutrients may be limiting crop productivity in many small scale farming systems, contributing to the current yield gaps (Lisuma et al., 2006, Van der Zaag, 2010). Indeed, strong response to the application of secondary and micronutrients was observed in the high and intermediate response classes (Fig. 6). These responses were not always accompanied by response to lime application and in these cases are assumed to indicate a deficiency of either/or S, Zn and B, which was the case for Kasungu, Pampaida and Mbinga. The results of the multivariate analysis indicates differences in micronutrient concentrations (Zn, B and also Mn) between the response classes, but at different levels of significance (Table 5). The poor non-responsive cluster, for example, had the lowest median values for Zn, B and Mn and even Cu. For instance, its median Cu of 0.8 ppm is within deficiency range being lower than the critical limit of 1 ppm (Lopes 1980). For the other response classes the median value is higher (though not significantly), but the lower range in all cases is below the critical value, indicating that Cu deficiency is also a likely widespread problem. Although studies involving Cu are rare within SSA, it was identified by Lisuma et al. (2006) as the limiting nutrient at Mpangala, Tanzania. Several fields mainly in the poor non-responsive and low responsive clusters had Mn concentration below the critical limit of 25 ppm reported for maize by Adeoye and Agboola (1985). Also for the intermediate response class, the lower range on Mn concentration is below the critical limit. Boron seems to be critically low for all the response classes with critical values indicated in literature ranging from 0.15 ppm to 0.5 ppm (see for example Aref, 2011), even though the high responsive cluster has significantly higher B values than the non- and low responsive soils. The analyses indicated that Zn was required to move the poor non-responsive cluster to responsive ones. However, in general Zn seems to be a less critical problem given that the median value for all response classes are above the critical values of 0.45 ppm to 1.17 ppm generally indicated in literature (Kumari et al., 2013, Patil et al., 2014). The poor non-responsive cluster had a significant lower Zn concentrations than any of the other response classes, though the values were within the critical limit. Previous studies have associated micronutrient deficiencies in SSA with infertile soils that have been subjected to long-term cultivation with addition of little or no fertilizer or organic nutrient resources (Zingore et al., 2008). The micronutrient deficiencies are not expressed for the non-responsive soils and at lower yield levels, because of other overriding constraints. Overall, there is a need for fertilizer recommendations that address the requirement for balanced fertilizer application, including micronutrients, under highly variable soil fertility conditions, and SSA needs to rise to this challenge if a green revolution is to be realized. Further research should indicate the extent to which B, Zn and Cu play a role in limiting response.

The occurrence of soils where crops do not show any significant response to fertilizer application could result from soil physical constraints that cause limited water availability and possibly restricted root development. Limited water holding capacity, poor infiltration rates, high surface runoff, and poor management practices may contribute to the limited availability of water to the crop, but these factors were not studied here and are therefore not discussed above.

### 4.3 Managing soils of different response classes

The management of soils in SSA requires a clear distinction between the intermediate and high response environments on the one hand and the poor low and non-responsive environments on the other hand. For the responsive environments the focus should be on optimizing management of inorganic nutrient inputs, including micronutrients, while maintaining soil organic matter management. In most current fertilizer recommendations in SSA, N and P are the nutrients of focus for cereal crops, but the significant maize yield response to micronutrients in the sites with the high and intermediate responsive soils suggests the need to take these nutrients into consideration (see also Vanlauwe et al., 2015). In all these cases the application of manure seems to improve crop performance.

Crop yield response to P application evident in all the sentinel sites (except Kibersahi and Kasungu; Fig. 3) indicates the importance for P fertilization. The widespread requirement for P is demonstrated by 6 out of the 9 sentinel sites having median available P levels below the critical value (15 mg kg^−1^; Nandwa and Bekunda 1998) of which 4 sites (Sidindi, Kontela, Koloko, and Pampaida) have median values of available P below 6 mg P kg^−1^, which is considered extremely low.

There was little variation in soil K levels between the various response classes, but K limitation was often expressed in responsive environments (the intermediate and high response classes), which explains why in Mbinga and Kasungu a clear overall effect of the omission of K is observed. For the other sites there was varied response to K, which seems to suggest that there are pockets where K is limiting. In sites like Sidindi, Kiberashi, Nkhata Bay and Thuchila it would be worthwhile to identify those pockets and address the K limitations to improve productivity.

With balanced application of N, P and K, yield levels of 4 t ha^−1^ can be achieved in case of the intermediate response class and 5 t ha^−1^ in case of the high response class (with good agronomic practice). But yields can be further increased to 5.5 t ha^−1^ and 6.5 t ha^−1^ respectively, with the proper input of organic resources and proper micro-nutrient application. A possible problem of soil acidity (in case of the intermediate response class) can be addressed with lime application, the application of manure or other measure to increase the pH and lower the Al concentration. Most sites require a combination of nutrients and manure and in specific cases lime to achieve attainable yield levels. Existing efforts to enhance fertilizer use to increase crop productivity should target the intermediate and high responsive soil classes, while aiming for balanced crop nutrition and putting measures in place to improve agronomic efficiency of the fertilizer application.

The poor non-responsive and low responsive soils are the most challenging and require specific management once the underlying causes are understood (Tittonell and Giller, 2013). Applying fertilizers and soil amendments to address those limitations is ineffective in the short-term. Rather attention should be devoted to restoring the productivity (responsiveness) of these soils through improved soil water management, and application of organic resources to increase SOC in the medium term, without expecting significant crop productivity improvement in the short-term. Restoring soil productivity may require application of large amount of manure for several years (Zingore et al., 2007), which in most cases is not practical for smallholder farmers in SSA. Better manure management (improved collection, storage), better integration of livestock (e.g. having pastures in the crop mixtures) and inclusion of agroforestry perennials in the system could significantly increase the amount of organic matter available to smallholders for restoring soil productivity. Changes of crops and in land use could also be considered depending on the identified limitation.

Major challenges remain on how to identify non-response soils at various spatial scales so that appropriate management practices for their rehabilitation can be effectively targeted. At scale, there could be scope for application of new technologies that are being developed for soil analysis, including infrared spectroscopy, mapping and surveillance (Shepherd and Walsh, 2007). This is increasing the feasibility for large-scale data collection for diagnosis of soil fertility constraints and improving targeting of technologies for increasing crop productivity in heterogeneous smallholder farming systems in SSA. Diagnostic trials, with treatments added to also diagnose constraints related to soil water availability, would be most useful to confirm the non-responsive and low-responsive status and to identify the constraints that cause the lack of response to nutrient application and soil amendments.

## 5 Conclusions

Diagnostic trial provide a relevant tool for yield gap assessment and provide data and information relevant for developing strategies and identifying possible solutions to improve crop productivity. A high degree of variability in crop response to nutrients and amendments is observed in major cereal growing areas in SSA, and this is associated with variability in soil characteristics within and between sites. The analyses of response patterns of crops to the various treatments in different fields allowed the grouping of fields into response classes. The problem of poor non-responsive and low-responsive soils is widespread and severe (approximately 50% of the cases), and if not corrected will limit the opportunities for sustainable intensification of agricultural smallholder production. Management of soil fertility through balanced crop nutrition that takes account of site-specific deficiencies in macro and micronutrients (however widespread) and considers the use of manure and other organic soil amendments is needed to achieve maize yields of over 4 t ha^−1^ on soil of the intermediate response class and yields of over 5 t ha^−1^ on soils of the high response category. In line with this, there is need to develop fertilizer formulations that address site-specific limiting nutrients. Research is needed to further establish crop response patterns and underlying characteristics, and to define the extent of micronutrient elements limitation to crops in SSA.

## Figures and Tables

**Table 1:** Locational attributes, number of fields, cultivar and selected biophysical characteristics of sites where field trials were conducted.

Site	Country	Latitude (Decimal degrees)	Longitude(Decimal degrees)	Elevation (m.a.s.l)	No. of fields	Year^a^	SR (mm)	Major farming system	Land form	Cultivar^b^
Koloko	Mali	12.5	−6.3	290	21	2009	810	Sorghum	Flat	Sorghum Kéninké (2.9 t/ha)
Kontela	Mali	14.8	−11.0	60	28	2009	565	Sorghum	Flat	Sorghum Séguifa Malisor 92-1 (2.4 t/ha)
Thuchila	Malawi	−15.9	35.3	710	21	2010	710	Maize/pigeonpeas	Flat	Maize SC403 (5.8 t/ha)
Kasungu	Malawi	−12.8	33.3	1060	24	2011	740	Maize	Flat	Maize DK8033 (11.6 t/ha)
Nkhata Bay	Malawi	−11.6	34.2	560	28+ 25	2010 and 2011	870	Cassava/maize	Hilly	Maize SC627 (7.5 t/ha)
Kiberashi	Tanzania	−5.3	37.5	1070	12	2010		Maize/pigeonpeas	Gentle slopes	Maize Pannar67 (6.7 t/ha)
Mbinga	Tanzania	−11.1	35.1	1000	31	2010	990	Maize	Hilly	Maize UH6303 (9.6 t/ha)
Sidindi	Kenya	0.1	34.4	1340	23	2010	750	Maize/beans	Flat to gentle slopes	Maize PH04 (12.1 t/ha)
Pampaida	Nigeria	11.3	8.2	600	29	2010	715	Maize/sorghum	Flat	Oba Super 2 (7.8 t/ha)

a indicates year when trial was established. SR = Seasonal Rainfall.

b data in brackets is attainable yield observed in the current trial for the indicated cultivar.

**Table 2:** Treatments implemented in AfSIS diagnostic trials.

Treatment	Description^a^
Co	Control: no nutrient added
NPK	Macronutrients added
−N	P and K applied (N omission)
−K	N and P applied (K omission)
−P	N and K applied (P omission)
+MN	NPK + Secondary and Micro-nutrients (CaMgSZnB) applied
*+MA*	NPK + manure applied
*+L*	NPK + lime applied

a nutrients were applied at 100 kg N ha^−1^, 30 kg P ha^−1^, and 60 kg K ha^−1^ for maize and 60 kg N ha^−1^, 20 kg P ha^−1^, and 30 kg K ha^−1^ for sorghum. Secondary and micronutrients, in the +MN, were applied at 10 kg Ca ha^−1^, 5 kg Mg ha^-1^, 5 kg S ha^−1^, 3 kg Zn ha^−1^ and trace amounts of B. Manure was applied at 10 t ha^−1^ on dry matter basis and lime at 500 kg ha^−1^.

**Table 3:** Major soil types and soil chemical and physical characteristics from diagnostic trial fields in different sites studied. Soil samples were obtained from the specific trial fields all at 0–20 cm depth before application of fertilizers and amendments.

Site	pH	Total C (%)	Total N (%)	Avail. P (ppm)	Phosphorus sorption index (PSI; meq/100 g)	Al (Mehlich 3; ppm)	Clay (%)	Sand (%)	Major soils
Koloko (Mali)	6.3 (5.4, 7.2)	1.5 (1, 3.3)	0.11 (0.07, 0.25)	5.6 (1.2, 35.4)	68 (35, 243)	704 (328, 1622)	42.3 (13.1, 87.5)	20.5 (2.4, 64.1)	Fluvisol
Kontela (Mali)	6.2 (5.3, 7.8)	0.79 (0.15, 0.82)	0.32 (0.01, 0.07)	3.6 (1.7, 26.1)	56 (42, 168)	460 (298, 696)	31.5 (15.5, 63.6)	41.1 (13.8, 65)	Arenosol
Thuchila (Malawi)	6.2 (5.7, 6.9)	0.72 (0.24, 2.24)	0.05 (0.01, 0.16)	26.1 (3.4, 151)	44 (7, 153)	663 (269, 1190)	36.8 (6.9, 81.1)	42.3 (8, 87.2)	Lixisols^b^
Kasungu (Malawi)	6.4 (5.4, 7.2)	0.6 (0.3, 1.5)	0.03 (0.02, 0.09)	52.8 (3.4, 360)	0 (−14, 67)	634 (267, 1140)	24.1 (15.8, 68.9)	56.8 (16.3, 69.3)	Luvisols and Gleysols^a^
Nkhata Bay (Malawi)	5.2 (4.1, 6.6)	1 (0.3, 3.5)	0.07 (0.01, 0.28)	22.1 (2.6, 354.2)	47 (−16, 296)	1118 (219, 1870)	35.5 (10.1, 72.7)	33.5 (5.1, 74)	Ferralsols^b^
Kiberashi (Tanzania)	6.1 (5.1, 7.2)	2.6 (1.4, 6.6)	0.19 (0.1, 0.54)	9.5 (2.5, 59.8)	39 (10, 128)	596 (253, 1050)	46.1 (14.6, 88.1)	38.3 (5.3, 73.9)	Luvisols^b^
Mbinga (Tanzania)	5.6 (4.5, 6.6)	1.6 (0.6, 4.8)	0.11 (0.05, 0.27)	9.9 (1.2, 83.8)	224 (115, 353)	1870 (1458, 2324)	80.2 (47.9, 90)	8.1 (4, 22.9)	Cambisols and Acrisols^b^
Sidindi (Kenya)	5.6 (4.9, 7.6)	1.3 (0.7, 2.2)	0.12 (0.05, 0.19)	3.6 (0.4, 81.2)	125 (40, 233)	1030 (654, 1570)	80.9 (47.1, 95.5)	5.4 (0.5, 38.1)	Ferralsols and Acrisols^b^
Pampaida (Nigeria)	6 (5.3, 7.4)	0.4 (0.3, 0.7)	0.03 (0.02, 0.04)	5.5 (2.2, 23)	27 (9, 50)	397 (300, 723)	17.7 (12.2, 43.9)	51.3 (28.3, 68.7)	Arenosol

Numbers are median with minimum and maximum in brackets.

a from Ngwira et al. (2012).

b from Harmonized World Soil Database accessed on 7th June 2013.

**Table 4:** Distribution of fields from each site in the various responsiveness clusters.

Site	Cluster 1a (poor Non-responsive fields)	Cluster 1b (fertile Non-responsive fields)	Cluster 2 (Fields responsive to N and P and to manure)	Cluster 3 (low response fields)	Cluster 4 (fields highly responsive to N)
Kasungu	0	0	6	6	12
Kiberashi	4	5	0	3	0
Mbinga	2	2	19	5	3
Nkhata Bay	21	0	16	14	2
Pampaida	1	0	22	4	2
Sidindi	4	1	6	10	2
Thuchila	8	0	0	12	1
Total	40	8	69	54	22

*Data in Nkhata Bay are from 2 seasons.

**Table 5:** Selected soil characteristics of the 4 derived clusters.

	Cluster 1a (poor Non-responsive fields)	Cluster 1b (fertile Non-responsive fields)	Cluster 2 (Fields responsive to N and P and to manure)	Cluster 3 (low response fields)	Cluster 4 (fields highly responsive to N)
pH	5.6 (4.3, 6.6)	6.1 (5.4, 7.1)	5.5 (4.1, 6.7)	5.7 (4.3, 6.7)	6.3 (4.6, 7.1)***###
%C	1.2 (0.7, 3.2)	2.1 (0.7, 5.2)	1.0 (0.2, 4.7)^a^##	1.5 (0.4, 4.0)	1.0 (0.4, 2.5)*###
Ca:Mg	2.56 (1.31, 3.99)	2.6 (1, 4.12)*	2.8 (1.02, 4.89)^b^	2.96 (1.25, 3.93)	4.5 (2.06, 6.82)***###
Na (ppm)	24 (6.9, 54.7)	26 (14, 70.5)	30 (8.9, 112)	31 (11, 188)	37 (15., 107)**#
P (ppm)	17 (1.8, 196)	11 (0.8, 40)	11 (0.2, 83)^b^	18 (1.3, 354)	46 (3.7, 360)
Al (ppm)	1040 (339, 2324)	816 (537, 1873)	1248 (408, 2315)**##	890 (253, 2157)	841 (441, 2113)^b^, ^a^
Mn (ppm)	94 (8, 568)	100 (15, 700)	210 (17, 836)^b^	130 (10, 667)^b^	159 (34, 630)
S	9.3 (5,17.6)^a^	7.9 (6.3,16.6)	9.4 (3.18,24)	8.5 (3.02,14.4)^b^	9.3 (4.71,36.2)*
B (ppm)	0.07 (0.001, 0.23)	0.34 (0.001, 1.25)**##	0.12 (0.001, 0.35)	0.1 (0.001, 0.42)	0.16 (0.001, 2.22)^b^, ^a^
Zn	1.81 (0.75,4.74)^a^	2.23 (0.41,8.72)	2.14 (0.43,10.2)^b^	2.31 (0.65,10.3)^b^	2.57 (0.67,20.6)*

Values are median. Values in brackets are minimum and maximum, respectively.

a indicates differences from Cluster 3, i.e., using the low responsive cluster as base category.

b indicates differences from Cluster 1a, i.e., using the poor non-responsive cluster as base category. # or * = P < 0.05, ## or ** = P < 0.01, ### or *** = P < 0.001. Critical limits for micronutrients (DTPA) are: 2 (lower) and 140 (upper) Mn (ppm); 4.5 (lower) Fe ((ppm; Sillampaa, 1982); 1 (lower) Cu (ppm; Lopes 1980).
